# Utility of Routine Outpatient Cervical Spine Imaging Following Anterior Cervical Corpectomy and Fusion

**DOI:** 10.7759/cureus.387

**Published:** 2015-11-23

**Authors:** Atman Desai, Arjun V Pendharkar, Jessica G Swienckowski, Perry A Ball, Scott Lollis, Nathan E Simmons

**Affiliations:** 1 Department of Neurosurgery, Stanford University School of Medicine; 2 Section of Neurosurgery, Dartmouth-Hitchcock Medical Center

**Keywords:** corpectomy, outpatient, radiograph, follow-up

## Abstract

Background: Construct failure is an uncommon but well-recognized complication following anterior cervical corpectomy and fusion (ACCF). In order to screen for these complications, many centers routinely image patients at outpatient visits following surgery. There remains, however, little data on the utility of such imaging.

Methods: The electronic medical record of all patients undergoing anterior cervical corpectomy and fusion at Dartmouth-Hitchcock Medical Center between 2004 and 2009 were reviewed. All patients had routine cervical spine radiographs performed perioperatively. Follow-up visits up to two years postoperatively were analyzed.

Results: Sixty-five patients (mean age 52.2) underwent surgery during the time period. Eighteen patients were female. Forty patients had surgery performed for spondylosis, 20 for trauma, three for tumor, and two for infection. Forty-three patients underwent one-level corpectomy, 20 underwent two-level corpectomy, and two underwent three-level corpectomy, using an allograft, autograft, or both. Sixty-two of the fusions were instrumented using a plate and 13 had posterior augmentation. Fifty-seven patients had follow-up with imaging at four to 12 weeks following surgery, 54 with plain radiographs, two with CT scans, and one with an MRI scan. Unexpected findings were noted in six cases. One of those patients, found to have asymptomatic recurrent kyphosis following a two-level corpectomy, had repeat surgery because of those findings. Only one further patient was found to have abnormal imaging up to two years, and this patient required no further intervention.

Conclusions: Routine imaging after ACCF can demonstrate asymptomatic occurrences of clinically significant instrument failure. In 43 consecutive single-level ACCF however, routine imaging did not change management, even when an abnormality was discovered. This may suggest a limited role for routine imaging after ACCF in longer constructs involving multiple levels.

## Introduction

Anterior cervical corpectomy and fusion (ACCF) is a relatively common procedure that may be performed for a range of indications, including spondylotic myelopathy or radiculopathy, trauma, infection, or tumor [1-5]. Routine postoperative radiographs are often obtained after this procedure, exposing patients to radiation and adding to the overall costs; yet, there are few data to support this practice. Previous studies have suggested that for anterior cervical discectomy and fusion (ACDF) performed for cervical spondylosis, routine postoperative radiographs in asymptomatic patients may not be warranted [6-7].

Cervical corpectomy, however, is a more complex procedure than ACDF, with increased stress often placed upon the arthrodesis and supporting instrumentation [8]. Some studies have reported an increased rate of graft displacement and pseudoarthrosis after ACCF when compared to ACDF [9-10]. Furthermore, ACCF may often be augmented with posterior stabilization involving long instrumented constructs. These factors suggest that ACCF, in theory, may warrant increased postoperative surveillance [11]. In addition, smoking, steroid use, previous cervical spine surgery, previous pseudoarthrosis, pre-existing deformity, and the indication for surgery may all influence the decision to obtain routine imaging [12-15].

While the above factors may, in theory, increase the risk of detecting construct failure, the actual utility of routine imaging also relates to its influence upon subsequent management. As such, it is also unclear how often unexpected findings on routine imaging after ACCF, in fact, lead to changes in management. The purpose of this study was to evaluate the utility of obtaining routine static x-rays after single or multiple level ACCFs are performed for a range of indications.

## Materials and methods

A retrospective analysis of the electronic medical record of all patients undergoing ACCF in our department from 2004-2009 was performed. Institutional Review Board approval was obtained for this study. Informed patient consent was obtained at the time of treatment. Patients undergoing a single or multilevel anterior cervical corpectomy, using a fibular allograft and/or iliac crest autograft, with or without a ventral cervical plate, with or without posterior augmentation, were all included in the study. All patients had postoperative radiographs within 24 hours of surgery and had routine clinical outpatient follow-up with further imaging scheduled for four to 12 weeks and thereafter on an as-needed basis, or per the surgeon’s typical follow-up pattern. Patient follow-up up to two years was analyzed.

The medical record was reviewed and patient baseline characteristics, operative detail, and postoperative course recorded. Baseline characteristics analyzed included patient age, sex, indications for surgery, preoperative smoking status, steroid use, and osteoporosis. Operative details included cervical levels and the number instrumented, type of graft used, and the presence of additional posterior fusion. Follow-up data reviewed included the use of postoperative steroids, outpatient appointment time from surgery, clinical findings, and radiographic findings. Where unexpected radiographic findings were noted, we also recorded whether or not the patient underwent any change in therapeutic management as a result of the findings. 

## Results

### Baseline characteristics

Sixty-five patients aged between 16 and 83 (mean 52.2, median 51) underwent surgery between 2004 and 2009 (Table 1). Eighteen patients were female. Ten patients were current smokers at the time of surgery, three patients had a history of previous cervical spine surgery, and one had a history of pseudoarthrosis. Six patients had preoperative kyphosis and seven patients had preoperative listhesis. Forty patients had surgery performed for spondylosis, 20 for trauma, three for tumor, and two for infection. Forty-three patients presented with myelopathy, 34 patients with radiculopathy, and 33 with axial neck pain.

**Table 1:** Baseline characteristics of patients undergoing ACCF

Baseline Characteristics	Number of Patients
Mean (median) Age	52.2 (51)
Sex (female)	18
Smoking	10
Previous cervical spine surgery	3
Preoperative kyphosis	6
Preoperative listhesis	7
Pathology​	
Degenerative	40
Trauma	20
Tumor	3
Infection	2
Clinical complaint​	
Myelopathy	43
Radiculopathy	34
Axial neck pain	33

### Surgical details

Forty-three patients underwent one-level corpectomy, 20 underwent two-level, and two underwent three-level corpectomy (Table 2). Fifty-three patients had a fusion using a fibular allograft, four using an iliac crest autograft, and eight using both fibular allograft and iliac crest autograft. Sixty-two of the fusions were instrumented using a plate, and 13 had additional posterior augmentation with lateral mass screws or interspinous wiring. All of the patients underwent plain x-rays within 24 hours of surgery. There were no abnormal findings in any of these radiographs.

**Table 2:** Number of levels and cervical levels operated upon

Number of Corpectomy Levels Performed	Number of Patients	Cervical Level (Numbers)
One-level	43	C4 (7); C5 (15); C6 (16); C7 (5)
Two-level	20	C4+5 (8); C5+C6 (11); C6+C7 (1)
Three-level	2	C4+C5+C6 (2)

### Follow-up and imaging findings

Eight patients were lost to follow-up. Fifty-seven patients had follow-up with imaging at four to six weeks postoperatively and further follow-ups at the surgeon’s discretion. Mean follow-up was 10.3 months. Eight patients were put on a course of postoperative steroids.

At the routine appointments at four to 12 weeks, 54 patients were imaged with x-rays, two patients with CT scans, and one patient with an MRI scan to assess for resolving infection. Fifteen patients reported clinical complaints at the early visit, but none was explainable by abnormal imaging. Of the 43 patients having undergone one-level ACCF, 38 reported no symptoms. Among all 54 patients, unexpected findings were noted in six cases. All of these patients had undergone instrumented fusion. All had improved overall clinically from baseline when assessed at this visit, although one had developed a deltoid weakness. One patient required intervention because of abnormal findings; this involved asymptomatic recurrent kyphosis with screw pullout following a two-level corpectomy, which required anterior release and posterior fusion with lateral mass screws and interspinous wiring. The other five patients, one of whom was a smoker, were successfully managed with observation.

Between three months and two years, only one patient demonstrated abnormal imaging findings and this patient required no further intervention. Of the overall patient cohort, five patients underwent further cervical spine surgery over the two years, but only one (at three months) was a revision surgery as a result of the failure of the initial surgery (Table 3).

**Table 3:** Characteristics of patients with abnormal imaging at 4-12 week follow-up

Patient	Age/Sex	Indication	Surgery	4-12 Week Imaging	Unexpected Findings	New Symptoms	Management
1	52M	Degenerative	C5-C6 Allograft/autograft +posterior fusion	XR	Kyphosis	None	Reoperation
2	48F	Degenerative	C6 Allograft	XR	Screw pullout	Deltoid weakness	Observation
3	69M	Degenerative	C4-C5 Allograft	XR	Screw pullout and break, telescoping of graft	None	Observation
4	62M	Degenerative	C4-C5 Allograft	XR	Screw pullout	None	Observation
5	64M	Degenerative	C6-C7 Allograft	XR	Screw pullout	None	Observation
6	47M	Tumor	C4-C5 Allograft/autograft	XR	Telescoping of graft	Worsened baseline hoarseness	Observation

## Discussion

Routine imaging after ACCF is commonly obtained at outpatient follow-up visits in asymptomatic patients [16]. The rationale for this practice is usually to evaluate for instrument failure, graft placement, and fusion status. The results of our study suggest that this practice can identify asymptomatic instances of hardware failure up to two years but that this rarely leads to a change in management. No patients with a single-level ACCF in our study had a change in management as a result of unexpected findings of instrument failure on routine imaging, and only one patient with a multi-level ACCF, in whom a recurrent asymptomatic kyphosis was found, required operative management. In addition, no patients with a history of smoking, steroid use, previous pseudoarthrosis, or surgery for trauma, infection, or neoplasm had routine imaging necessitating operative management. 

The above results would suggest a limited role for routine imaging after ACCF. A previous study by Ugokwe, et al. [7] suggested that routine imaging after a single-level ACDF for degenerative disease was likely unwarranted and that significant instrumentation failure requiring intervention would be symptomatic. A more recent study by Shau, et al. [17] attempted to analyze the utility of postoperative radiographs in cervical spine fusion and found that in 140 postoperative outpatient visits for 43 patients following ACCF, none had imaging findings in clinic necessitating operative management, although the number of corpectomy levels was not reported. They also found no significant difference in the propensity for clinically significant abnormal imaging based on the surgical indication. ACCF comprises a more complex surgical procedure than single-level ACDF, with potentially greater stresses placed upon the construct and, therefore, increased likelihood of instrument failure. This likelihood may also in theory be affected by indication for surgery (e.g. trauma) and factors such as previous surgery, posterior instrumentation, smoking, and steroid use [11-15]. Our study found, however, that no patients following one-level ACCF had imaging findings requiring intervention regardless of the indication for surgery and also when the above factors were present. In our study, one patient, following a two-level corpectomy, was found to have an asymptomatic return of kyphosis on routine x-ray and required reoperation, suggesting that routine imaging may have a greater role for surveillance of larger constructs.  

Delivering quality in surgical care depends upon achieving good outcomes in a cost-effective manner. Central to this is the avoidance of unnecessary diagnostic or therapeutic procedures that expose patients to risk and add to overall cost. In light of this, several recent studies have attempted to address the utility of routine imaging after cervical spinal instrumentation and have tended to conclude that imaging may be best reserved for those patients who are symptomatic [6-7, 17]. Our own findings would support this recommendation in single-level ACCF, where those occasions with abnormal imaging were apparently not clinically significant and did not require a change in management. The patient in our series, however, who had undergone a multilevel corpectomy and in whom an asymptomatic kyphosis requiring reoperation was discovered on routine imaging may be part of a patient subset with longer constructs that would be an exception to such a rule. In essence, however, avoiding x-rays on the 38 out of 43 patients in our series who were asymptomatic would, in addition to avoiding exposure of patients to unnecessary radiation, have saved costs incurred by imaging, radiographic interpretation, and provider clinic visits.

The present study has several limitations that should be considered. It is retrospective in nature, with post hoc data collection and interpretation susceptible to bias. The study also represents the experience of a single institution, and it remains unclear whether these findings apply to other centers. In addition, the study size is relatively small and may be underpowered to detect a higher rate of abnormal radiographs that influenced management.       

## Conclusions

Routine imaging after ACCF can demonstrate asymptomatic instances of instrument failure. In forty-three consecutive single-level corpectomies, however, routine imaging did not change management, even when abnormalities were discovered. This may suggest a limited role for routine imaging after ACCF and encourage more judicious use.
